# A person-centered needs-tailored recovery program for community-dwelling people diagnosed with mental illness

**DOI:** 10.1192/j.eurpsy.2022.1601

**Published:** 2022-09-01

**Authors:** W.I. Liu, Y.-M. Tai, W.-L. Hsien

**Affiliations:** 1 National Taipei University of Nursing and Health Sciences, College Of Nursing, School Of Nursing, Taipei, Taiwan; 2 Tri-Service General Hospital Beitou Branch, Psychiatry, Taipei, Taiwan; 3 National Taipei University of Nursing and Health Sciences, School Of Nursing, Taipei, Taiwan

**Keywords:** person-centered care, Recovery, Randomized Controlled Trial, needs-tailored

## Abstract

**Introduction:**

The recovery of community-dwelling people diagnosed with mental illness is positively correlated with having their needs met; however, only a few person-centered services provide solutions that are tailored to the needs of such populations.

**Objectives:**

The aim of this study was to evaluate the effectiveness of a needs-tailored recovery program.

**Methods:**

A double-blind randomized controlled trial was used. In the experimental group, people diagnosed with mental illness received homecare services for six months as part of a new needs-tailored recovery program. The control group received existing community homecare services. Data were collected before and after the intervention (July 2020 to January 2021). The primary outcome was recovery, and secondary outcomes were needs, hope, empowerment, psychotic symptoms, and medication adherence.

**Results:**

The recovery program integrated the evidence-based care elements for community-dwelling people diagnosed with mental illness that we had identified: need satisfaction, hope, empowerment, and medication adherence. In total, 62 participants were included. There were no significant pre-test differences between the two groups in terms of demographic or baseline variables. However, there were significant differences between them in the extent of improvement in recovery, needs, hope, and empowerment, and medication adherence improved significantly but similarly in both groups.

**Conclusions:**

Our person-centered recovery program fitted individuals’ needs and improved recovery and related elements for personal recovery among community-dwelling people diagnosed with mental illness. This study increases our understanding of recovery-oriented care to prioritize therapeutic alliance, integrated needs assessment, individualized unique goals, hope, and empowerment.

**Disclosure:**

No significant relationships.

